# Outcomes of One-Anastomosis Gastric Bypass Conversion to Roux-en-Y Gastric Bypass for Severe Obesity: A Systematic Review and Meta-analysis

**DOI:** 10.1007/s11695-023-07050-y

**Published:** 2024-01-20

**Authors:** Narek Sargsyan, Bibek Das, Henry Robb, Christopher Namgoong, Iihan Ali, Hutan Ashrafian, Samer Humadi, Anuja Mitra, Matyas Fehervari

**Affiliations:** 1 Department of Surgery and Cancer, Imperial College London, London, UK; 2 Ashford and St. Peter’s Hospitals NHS Foundation Trust, Surrey, UK; 3 Department of Bariatric Surgery, Chelsea and Westminster Hospital, London, UK

**Keywords:** OAGB, RYGB, Revisional Bariatric Surgery

## Abstract

One-anastomosis gastric bypass (OAGB) is an effective procedure to treat severe obesity. However, conversion to Roux-en-Y gastric bypass (RYGB) is increasing. We therefore conducted a systematic review to determine the safety and efficacy associated with OAGB-RYGB conversion. A systematic search was conducted by three independent reviewers using Medline, Embase, and the Cochrane library following PRISMA guidelines. Six studies including 134 patients were selected who were undergoing OAGB-RYGB conversion. The most common indications were reflux (47.8%), malnutrition (31.3%), and inadequate weight loss (8.2%). Study outcomes demonstrated 100% resolution of bile reflux. Overall, there was medium-term weight gain of 0.61 BMI. OAGB to RYGB conversion leads to resolution of reflux symptoms. However, it is associated with weight regain, albeit this may be acceptable to patients to treat biliary reflux.

**Supplementary Information:**

The online version contains supplementary material available at 10.1007/s11695-023-07050-y.

## Introduction

Obesity remains a major health problem worldwide and bariatric surgery is the most effective treatment for severe obesity. One-anastomosis gastric bypass (OAGB) also known as the mini-gastric bypass has long been reported as a safe and effective procedure in the treatment of severe obesity and its associated medical problems [1, 2]. Laparoscopic Roux-en-Y gastric bypass (LRYGB) is considered as the gold standard. However, studies have demonstrated that OAGB is a safe and technically simpler alternative which can lead to more weight loss [3, 4].

Despite significant weight loss and resolution of weight-associated medical problems since its implementation, OAGB may expose patients to certain complications for which revisional bariatric surgery (RBS) may be necessary. RBS may be indicated for perioperative complications as well as for adverse effects occurring several years after OAGB. The most common reasons for revision after OAGB include bile reflux, persistent marginal ulcer, and malnutrition with variable reported conversion rates between 0.9 and 5.2% [5–8]. In such cases, OAGB can be reversed to normal anatomy or converted to another procedure, such as RYGB, with a conversion rate of 4.1% [8–10]. This has proven to be technically feasible for most cases and is associated with a moderate risk of perioperative complications [11]. However, the current literature describing conversion of OAGB to RYGB is still limited depending on the reasons for conversion. Therefore, we conducted a systematic review to determine the weight outcomes, safety, and efficacy associated with OAGB-RYGB conversion surgery.

## Methods

This systematic review was performed in accordance to a registered protocol and reported according to PRISMA (Preferred Reporting Items for Systematic Reviews and Meta-Analyses) [12]. The review was also registered on PROSPERO Centre for Reviews and Dissemination (registration number CRD42022379759).

A literature search was performed in October 2023 using MEDLINE (via PubMed), EMBASE (via OVID), and Cochrane database using MeSH terms in all combinations: “bariatric surgery” or “metabolic surgery” or “weight loss” or “obesity surgery” and “one anastomosis gastric bypass” or “mini gastric bypass” or “Roux-en-Y Gastric Bypass” or “Revisional Bariatric Surgery” or “conversion to Roux-en-Y” or “one anastomosis gastric bypass to roux-en-y.” Studies identified from the search strategy were entered into Covidence (Victoria, Australia) for duplication removal and bibliographic management. Three reviewers independently identified relevant studies and discrepancies were resolved by consensus between all authors. The exact search strategy is outlined in supplementary material (Sup.1)

### Inclusion and Exclusion Criteria

The inclusion and exclusion criteria were defined before commencement of the literature search. The following criteria were required for inclusion in the study:

(i) Randomised controlled trials (RCT), prospective or retrospective cohort studies.
(ii) Reported outcome of interest including bile reflux, malnutrition, inadequate weight loss.
(iii) Original, full publications published in the English language

All studies reporting on weight loss outcome data from OAGB to RYGB revisional bariatric surgery were included. Studies were excluded from analysis if no post-conversion weight outcome data relative to the pre-conversion baseline was reported. Cross-sectional studies as well as case control studies were also excluded.

### Data Extraction and Quality Assessment

A standardised data extraction form was developed on Covidence and two authors (BD and MF) independently extracted all relevant data. All discrepancy was resolved by group discussion. Quality scoring of studies was performed using the Newcastle-Ottawa assessment tool. The scale is divided into three broad stratifications: selection (consists of four items), confounder (including one item), and exposure (contains two items), with a total maximum score of 9 [13].

### Statistical Analysis

Data analysis was performed using Stata Software, Version 15.1. StataCorp LCC, TX. Random-effects analysis was used to calculate weighted mean difference and mass effect. All studies were included in the analysis if relevant data was available. Data was analysed using a random effects model and statistical heterogeneity was calculated using *I*^2^. An *I*^2^ of <30 was considered as low, 30–60 as moderate, and >60 as high heterogeneity. Results were computed and represented on forest plots (see supplemented section).

Our management of missing data in this meta-analysis includes multiple imputation approaches. Additionally, we applied non-classical approaches of ratio-of-means previously applied for combining non-classical sources.

## Results

**Fig. 1:**
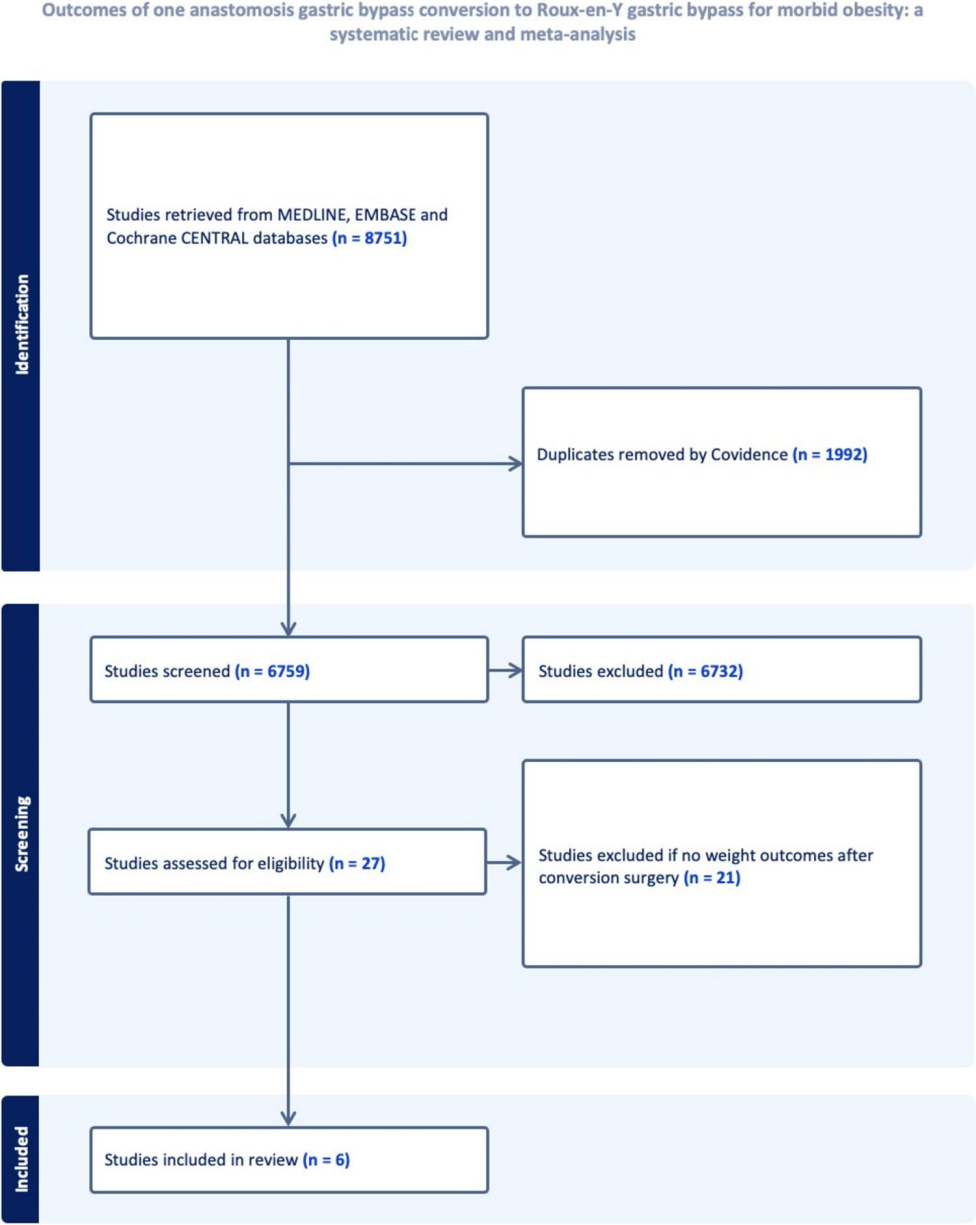
PRISMA outcome for OAGB conversion to RYGB

Six studies were found to fulfil the inclusion criteria and included in this systematic review, producing a pooled patient population of 134 patients who had undergone OAGB-RYGB conversion surgery (Fig. 1).

All studies were retrospective analyses of prospectively kept databases. The average quality of studies according to the Newcastle-Ottawa scale was good (8.0 (minimum 7 and maximum 9)) The most common point deduction was for adequacy of follow-up for cohorts (see Sup. Table2). The mean follow-up period ranged from 6 to 60 months. The mean age of patients was 45.9 years old (SD 3.6).

Patient’s baseline BMI were 42.6 kg/m^2^ (SD 4.3) prior to OAGB and 30.2 kg/m^2^ (SD 2.85) at conversional surgery. This study found a weight loss in means of %TWL of 35.49 (SD 1.56) and % EBMIL of 84.07 (range 11.04) following OAGB.

**Fig. 2:**
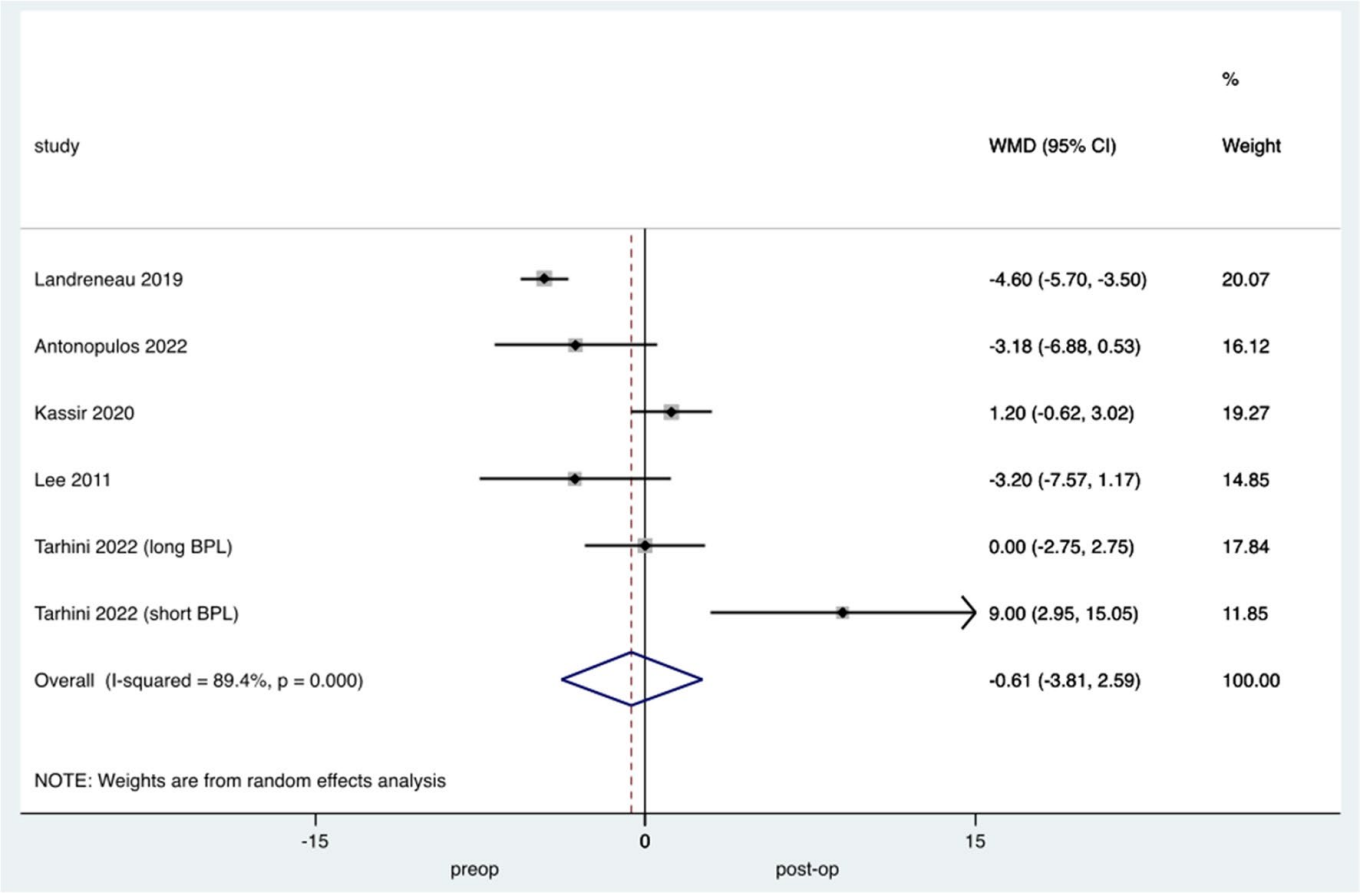
Forest plot demonstrating changes in BMI pre and post OAGB-RYGB conversion

Studies reporting BMI at >24 months following conversion reported overall significant medium-term weight regain (weighted mean BMI difference 0.61 (95% CI −3.81 to 2.59 *p*<0.005) with high study heterogeneity (*I*^2^ = 89.4%) (Fig. 2).

**Fig. 3:**
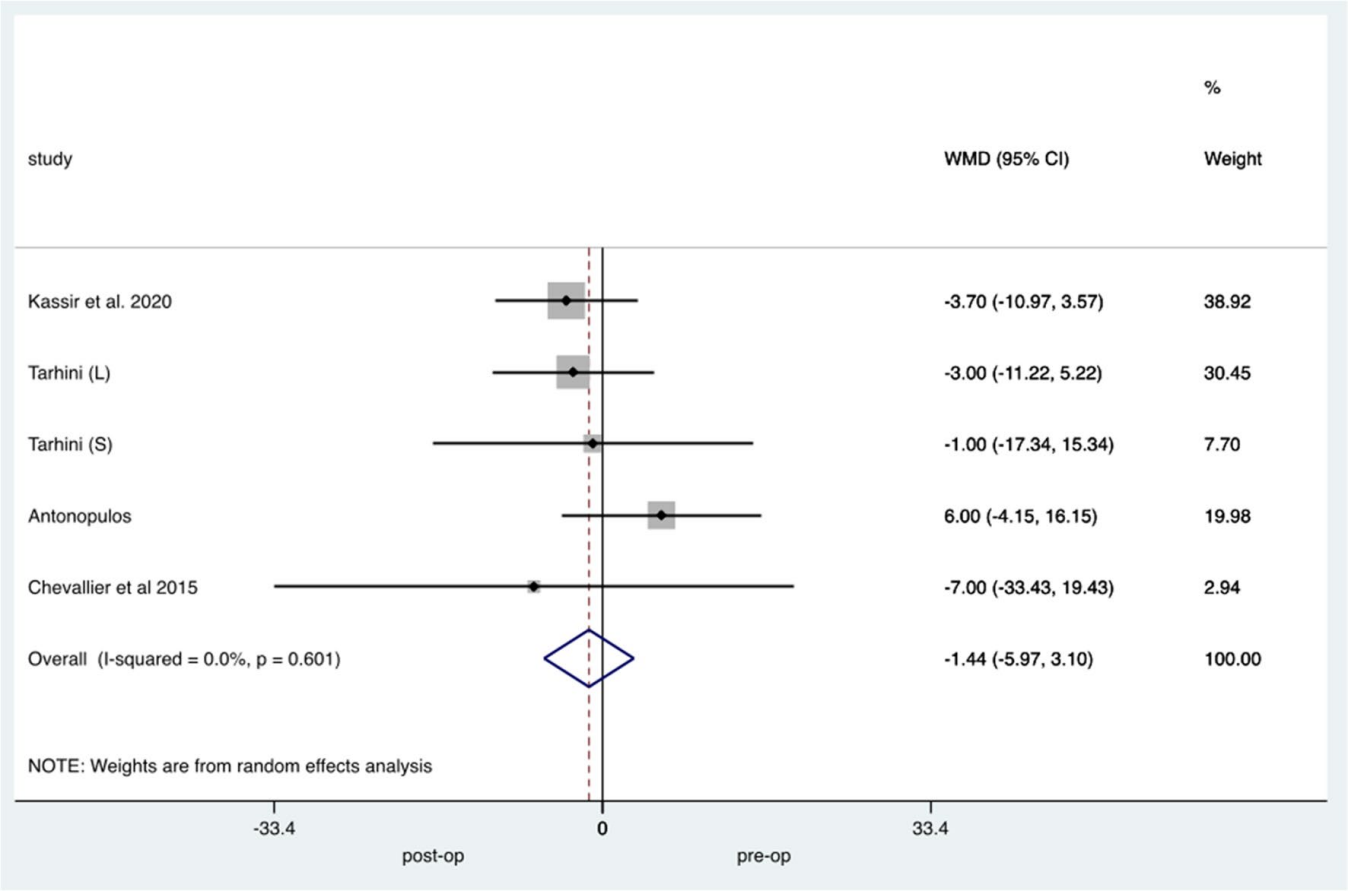
Forest plot demonstrating changes in weight pre and post RYGB conversion

Five studies including 116 patients reported on pre-RYGB and post-RYGB weight change; pooled analysis demonstrated a weighted mean increase of 1.44 kg in weight (95% CI −5.97 to 3.09, *p*=0.6) with low study heterogeneity (*I*=0) (Fig. 3).

The most common indications for conversion surgery were reflux (47.8%), malnutrition (31.3%), and inadequate weight loss (8.2%). Less common indications included nausea and vomiting (6%), weight regain (2.2%), gastrogastric fistula (3%), and anastomotic leak (1.5%). The afferent limb length for OAGB ranged from 150 to 200 cm. Conversion to RYGB Biliopancreatic limb length ranged from 50 to 200 cm. Alimentary limb length ranged from 50 to 150 cm.

**Fig. 4:**
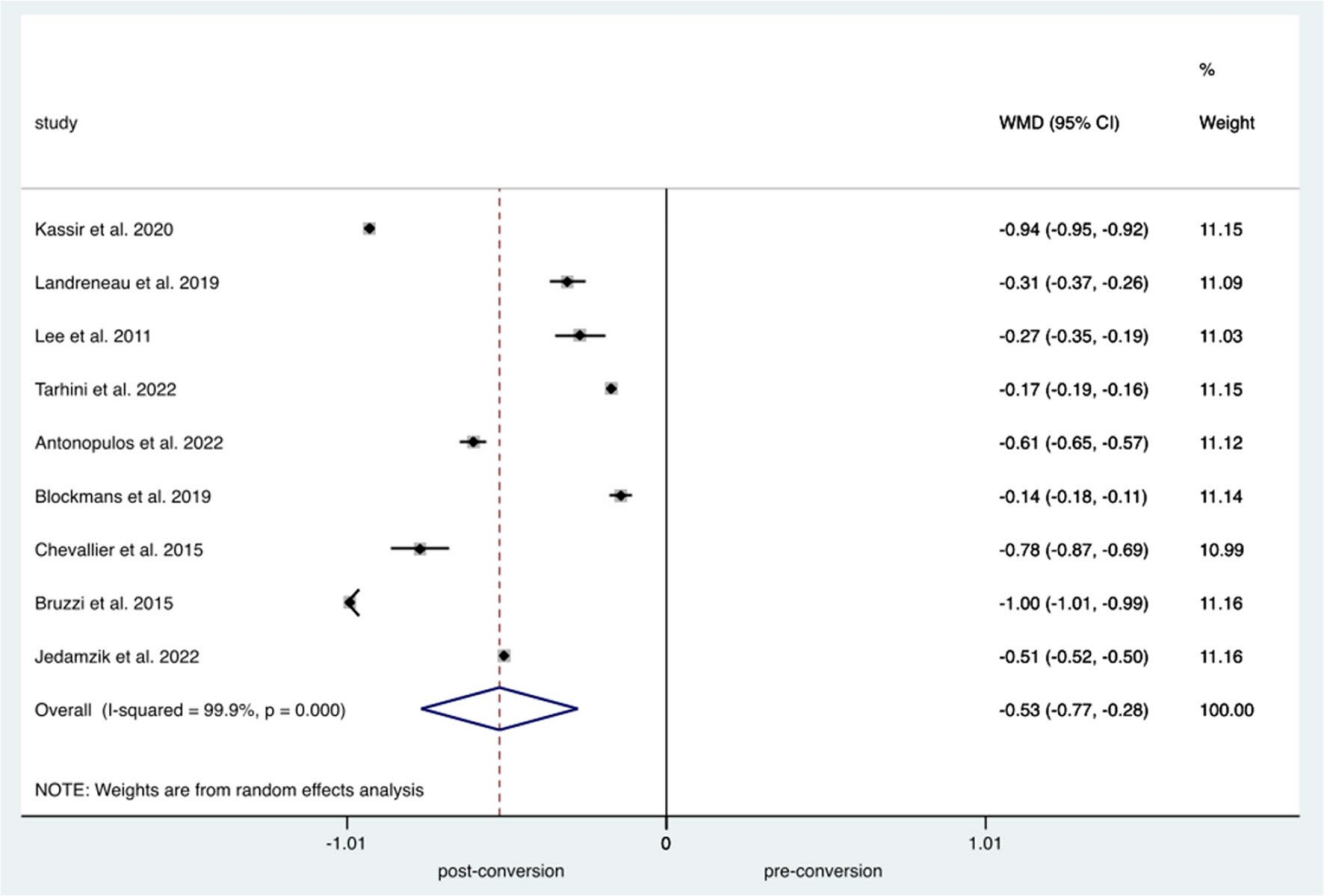
Forest plot demonstrating bile reflux pre and post OAGB-RYGB conversion

Comparing the pooled incidence of bile reflux before and after conversion to RYGB demonstrated a significant improvement in the incidence of bile reflux (*p*<0.001; WMD=−0.526; 95% CI −0.774 to −0.279; *I*^2^=99.9%) (Fig. 4). The weighted mean of resolution of biliary reflux symptoms suggested that almost 100% of the cases improved (95% CI 99–100%, *I*^2^=50.4).

**Fig. 5:**
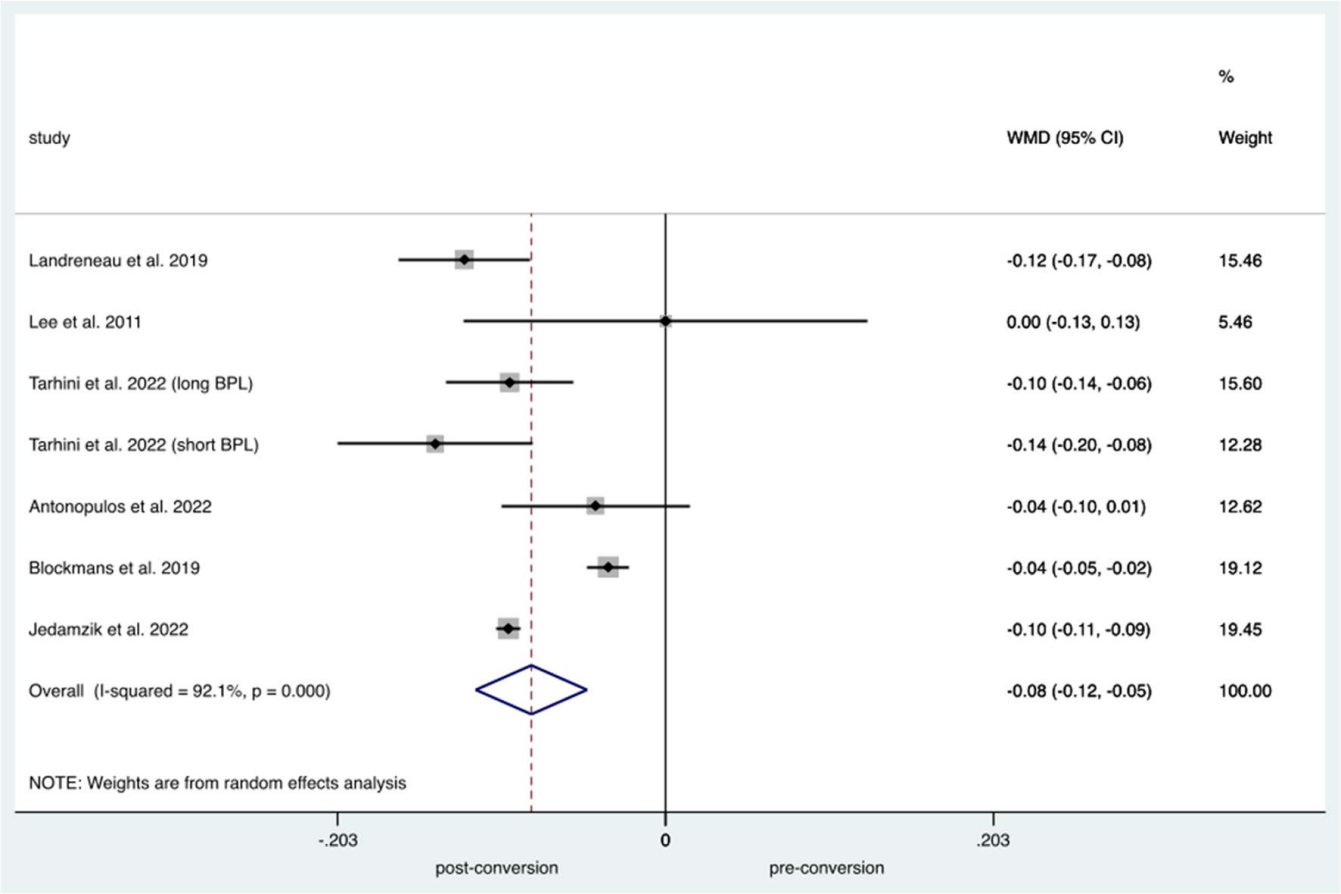
Forest plot demonstrating malnutrition pre and post OAGB-RYGB conversion

Random effects analysis of malnutrition pre- and post-conversion to RYGB demonstrated a significant difference in terms of resolution of malnutrition post-RYGB *p*<0.005 with a high interstudy heterogeneity (WMD: −0.083, 95% CI −0.118 to −0.049, *I*^2^=92.1%) (Fig. 5). The weighted mean of resolution of malnutrition symptoms demonstrated that almost 52.7% of the cases of malnutrition fully resolved (95% CI 0.148–0.907%, *I*^2^=100%).

**Fig. 6:**
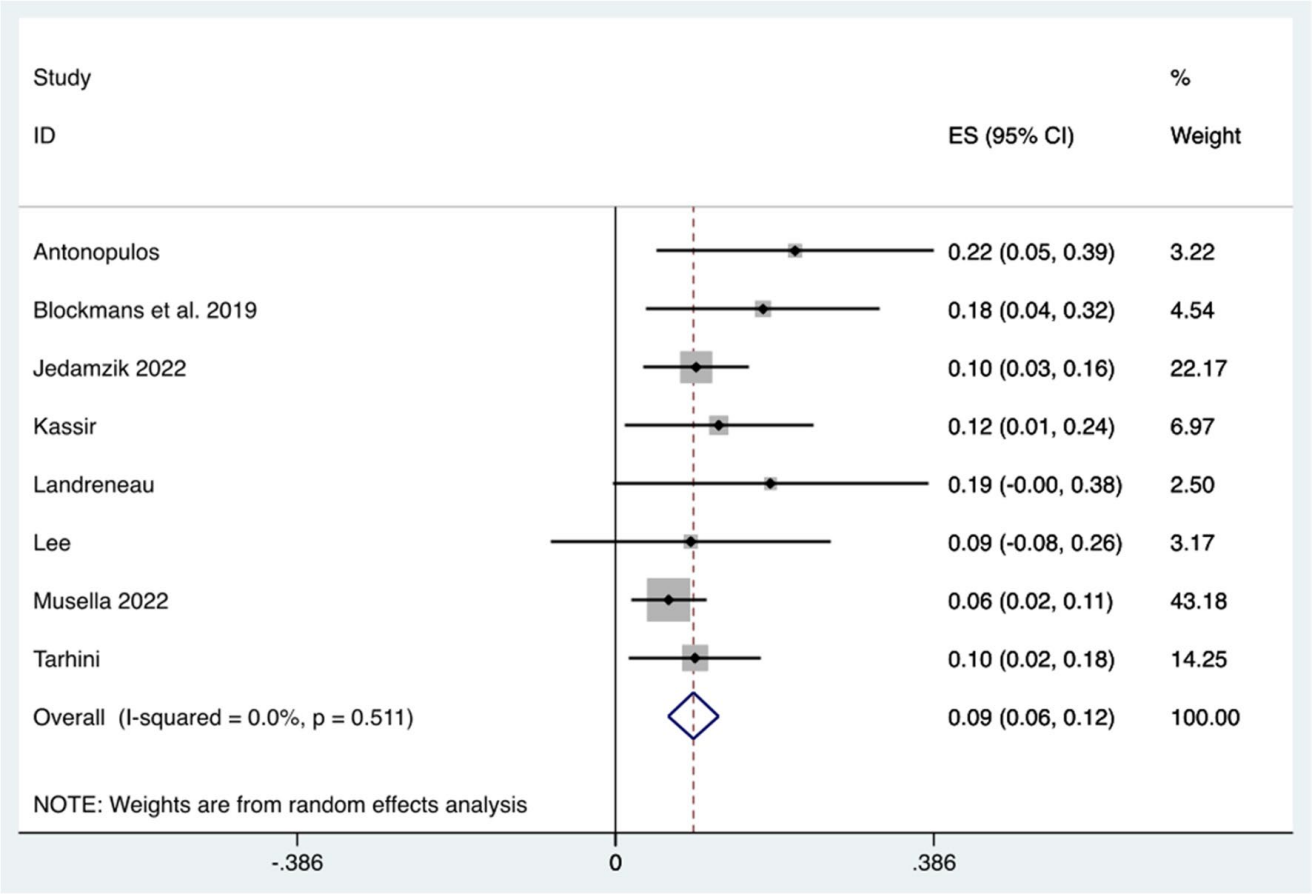
Forest plot demonstrating the weighted mean rate of major (Clavien-Dindo grade >3) post-conversion complications

The weighted mean rate of major (Clavien-Dindo grade >3) post-op complications was 9.4% (95% CI 6.4–12.5, *p*<0.005) (Fig. 6). There were no reported deaths after conversion surgery and the weighted mean time to conversion was 31.5 months.

**Fig. 7:**
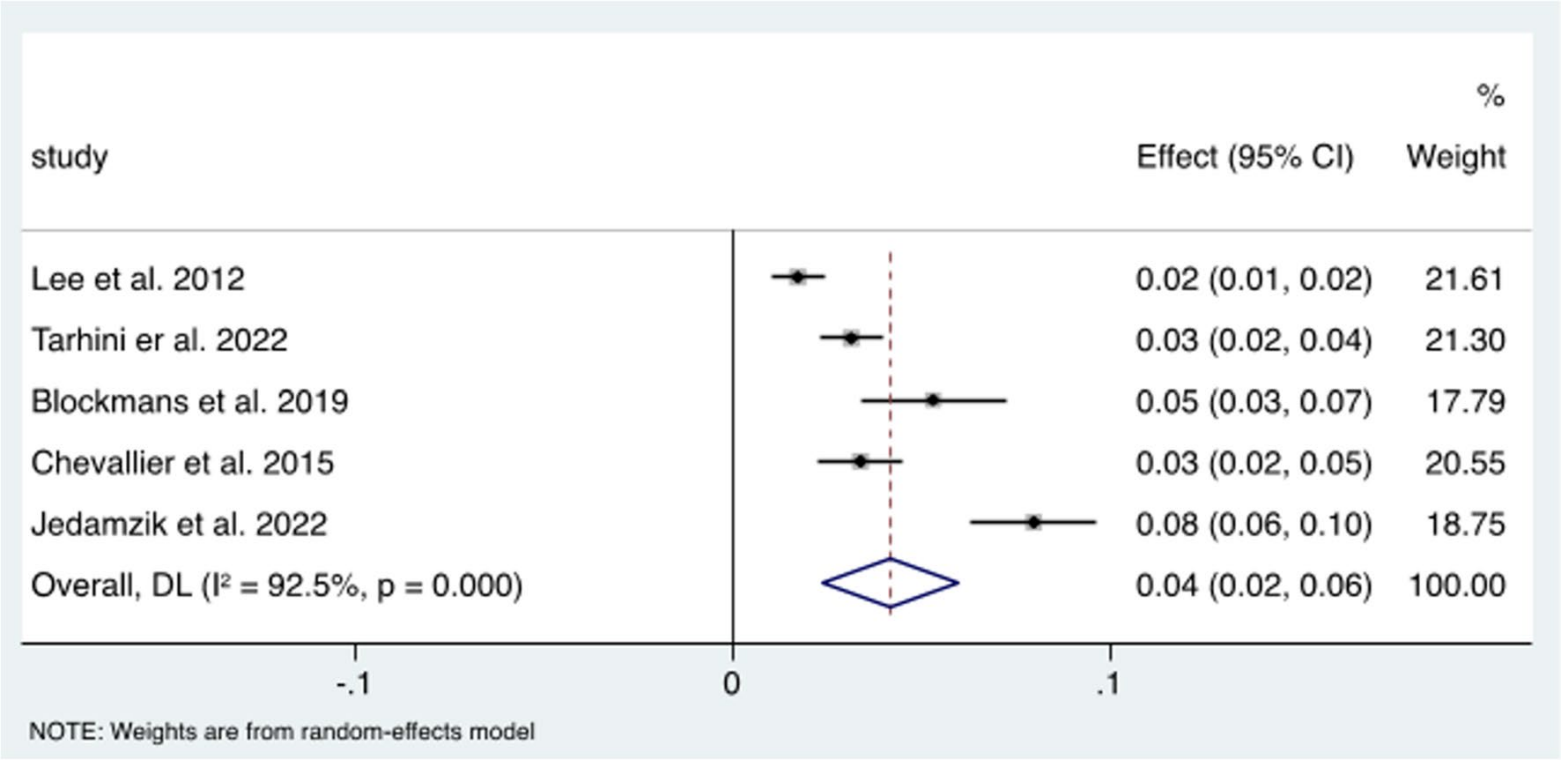
Forest plot demonstrating weighted mean analysis of OAGB conversion to RYGB

The weighted mean rate of conversion from OAGB to RYGB was found to be 4.2% (95% CI 2.4–6, *p*<0.005) with a high interstudy heterogeneity (*I*=92.5) (Fig. 7).

## Discussion

OAGB is the third most common bariatric surgical procedure worldwide with excellent weight loss data reported by several studies as well as a good safety profile as primary metabolic surgery [14]. Although bariatric surgery represents the only long-term effective treatment of severe obesity, revision procedures are occasionally indicated in patients experiencing post-bariatric surgery complications or failure to lose weight at different stages of follow-up. The revision rate after OAGB is reported as 4.2% which is similar to that after RYGB [15]. Revision rate after OAGB to normal anatomy is reported as 1%, with the most common indication being severe malabsorption, and is associated with a complication rate of 10.9% [16].

Several long-term complications specific to OAGB have been described that may warrant revisional surgery. Our study demonstrated that the most common indication for conversion surgery was biliary reflux (47.8%). This is well documented in literature and the theory is attributed to the surgical technique. In OAGB, there is a lack of anatomical barrier (sphincter) and the gastric pouch is permanently exposed to bile flow. This theory is in keeping with patients who suffer from insufficient lower oesophageal sphincter and may explain the lack of effectiveness of PPIs in OAGB patients. Other possible theories include the larger volume of the gastric pouch after OAGB (25–35 mL) in comparison to RYGB (15–20 mL) over time promoting dilatation and food stagnation and acid production. In addition to dilatation, impaired emptying of the gastric pouch and pre-existing or postoperative lower oesophageal sphincter insufficiencies and bile reflux can be a disabling digestive disorder to manage [17–19]. Our pooled analysis demonstrated that almost all diagnoses of bile reflux resolved after revision from OAGB to RYGB.

Several authors have reported that OAGB has a greater weight loss efficacy than RYGB which is attributed to its longer biliopancreatic limb and comparatively higher malabsorption [20]. The greater weight loss in OAGB patients prior to revisional surgery may possibly be explained by ongoing gastrointestinal symptoms such as GI symptoms from bile reflux or ulcers leading to patients refraining from food intake as well as ongoing malnutrition.

After revisional surgery to RYGB, there was a significant medium-term weight gain (0.61 BMI gain). The potential explanation of these results could be the longer exposure time of nutrients in the small intestine. However, a revision to L-BPL-RYGB is associated with less weight regain as reported by Tarhini et al., although it is technically a more challenging procedure and less effective against acid reflux than S-BPL-RYGB. The potential weight gain may be acceptable by some patients undergoing revisional surgery in order to improve bile acid reflux which was found to be the most common indication for revision from OAGB to RYGB. Lee et al. reported that patients who underwent revisional surgery for lack of weight loss from OAGB underwent repeat revisional surgery because of repeat lack of weight loss and weight gain [21]. This leaves the question whether patients who are resistant to weight loss from OAGB require exploration of other factors rather than be an indication for revisional bariatric surgery. Although many authors identify lack of weight loss or weight regain as the cause of bariatric surgery failure in the incorrect technique/surgery choice, no data is available on this topic and there is a small percentage of individuals refractory to bariatric surgery, regardless of the surgical treatment performed.

RYGB should not be offered for weight regain and patients should be counselled appropriately before conversion to make sure they understand they are not having another weight loss (bariatric) surgery but rather a corrective operation to deal with complications of OAGB.

Interestingly, Tarhini et al. report an observed difference in the improvement rate of bile acid reflux between long biliopancreatic limb (L-BPL-RYGB), this procedure consists of preserving the gastric pouch, gastrojejunal anastomosis, and the entire length of the biliopancreatic limb (BPL) which is anastomosed 70 cm distally to the gastrojejunal anastomosis; in comparison to a short BPL-RYGB (S-BPL-RYGB), which consists of resecting the gastrojejunal anastomosis, shortening the gastric pouch and performing a RYGB with a 150-cm-long alimentary limb and short 50-cm-long BPL. Overall S-BPL-RYGB led to significant improvement in bile acid reflux, malabsorption, and diarrhoea. However, the S-BPL-RYGB was also associated with greater weight regain; this can be explained with greater food contact with the intestine and greater absorption [22]. However, we have not noted this trend in other studies. A meta-analysis by Kamocka et al. (2022) assessing the difference in outcomes between short PBL and long BPL in RYGB found no significant difference in weight change [23]. There are several surgical methods when performing a RYGB, though no significant differences in weight loss between the different methods have been reported [24].

The changes may be explained by the metabolic modifications introduced by the revisional surgery described as BRAVE effect (bile flow alterations, reduction of gastric size, anatomical gut rearrangement and altered flow of nutrients, vagal manipulation, enteric gut modulation) [25, 26]. OAGB to RYGB rearranges the anatomy leading to changes in bile flow and resolution of reflux. Anatomical rearrangement of the gut from OAGB to RYGB leads to improvement in malabsorption as it leads to greater food contact with the intestine with greater absorption; however, this also leads to a small amount of weight regain after conversion surgery. Rearranging the anatomy of the gastric pouch may lead to further vagal manipulation leading to the weight gain observed. However, the changes observed could also be due to an anomalous metabolic pathway, not altered by the BRAVE effect. These effects may offer a paradigm to identify the profound mechanisms driving bile reflux, malnutrition, and gut microbiome [27, 28].

### Strengths and Limitations

The strength of this study includes comprehensive data collection involving extensive data published in the literature and rigorous data extraction and robust statistical analysis of relevant data. Limitations of this study include a small number of patients due to a rare condition, considerable heterogeneity in the reported outcomes that may be due to heterogeneity of eligibility criteria, and patient demographics among the mainly non-randomised observational studies. Another factor may be the lack of robust quality assurance of the procedures being performed which is an essential component of interventional-based trials.

## Conclusion

The results of this study have demonstrated that revisional surgery from OAGB to RYGB is an effective treatment in patients with post-OAGB refractory biliary reflux which was the most common indication for OAGB revision to RYGB leading to complete resolution of symptoms. However, it is associated with weight regain, albeit this may be acceptable to patients to treat biliary reflux as the weight gain is small over medium term. Alternative options for revisional surgery should be explored for patients who have failed to lose weight after OAGB as conversion to RYGB may lead to further weight gain. Hence, this data does not suggest that performing a RYGB for failure to lose weight or weight would be recommended.

### Supplementary Information

ESM 1 (DOCX 15 kb)
