# Supplementary material for: Outcomes of One-Anastomosis Gastric Bypass Conversion to Roux-en-Y Gastric Bypass for Severe Obesity: A Systematic Review and Meta-analysis
Source: Obes Surg. 2024 Jan 20;34(3):976–84. doi: 10.1007/s11695-023-07050-y (PMC10899303; doi:10.1007/s11695-023-07050-y)
Supplement: Supplementary file 1 — (DOCX 15 kb) [file 11695_2023_7050_MOESM1_ESM.docx]

*Supplementary Material*

Table 1. Studies reporting on conversion of OAGB to RYGB: Study characteristics

| **First author and year** | **Study design** | **Sample size** | **Study quality score** | **Age (mean)** | **Pre OAGB BMI** | **Pre-RYGB conversion BMI** | **Post-RYGB revision BMI** | **Mean time to conversion (months)** | **RYGB Biliary Limb length** | **Length of Follow up post**  **Conversion (months)** |
| --- | --- | --- | --- | --- | --- | --- | --- | --- | --- | --- |
| Kassir et al. 2020 [12] | Retrospective | 32 | 9 | 45.6 | 40.1 | 26 | 27.2 | 18.3 | 150cm | 47.6 |
| Jedamzik et al. 2022 [13] | Retrospective | 75 | 8 | 47 | 44.4 | 29.8 | 29.1 | 29.1 | 35-100cm | 28.3 |
| Landreneau et al. 2019 [14] | Retrospective | 16 | 7 | 40.2 | x | 30.7 | 30.9 | 24 | 50cm | 10.2 |
| Lee et al. 2011 [15] | Retrospective | 11 | 8 | x | x | 29.2 | 26.0 | 34.8 | 50-100cm | 24 |
| Tarhini et al. 2022 [16] | Retrospective | 52 | 8 | 52 | 44 | 29 | 29 | 50 | 50cm & 200cm | 24 |
| Antonopulos et al. 2022 [17] | Retrospective | 23 | 8 | 42 | 42.6 | 28.0 | 28.7 | 34 | 70cm | 24 |

Table 2. Newcastle-Ottawa Score: Quality assessment of selected studies.

|  | Kassir et al. 2020 | Jedamzik et al. 2022 | Landreneau et al. 2019 | Lee et al. 2011 | Tarhini et al. 2022 | Antonopulos et al. 2022 |
| --- | --- | --- | --- | --- | --- | --- |
| 1. Selection |  |  |  |  |  |  |
| 1.Representativeness of the exposed cohort | 1 | 1 | 1 | 1 | 1 | 1 |
| 2.Selection of the non-exposed cohort | 1 | 1 | 1 | 1 | 1 | 1 |
| 3.Ascertainment of exposure | 1 | 1 | 1 | 1 | 1 | 1 |
| 4.Demonstration that outcome of interest was not present at start of study. | 1 | 1 | 1 | 1 | 1 | 1 |
| 1. Compatability |  |  |  |  |  |  |
| 1.Compatability of cohort on the basis of design analysis | 2 | 2 | 2 | 2 | 2 | 2 |
| 1. Outcome |  |  |  |  |  |  |
| 1.Assessmnet of outcome | 1 | 1 | 1 | 1 | 1 | 1 |
| 2.Was follow up long enough for outcomes to occur | 1 | 1 | 0 | 1 | 1 | 1 |
| 3.Adequacy of follow-up of cohorts | 1 | 0 | 0 | 0 | 0 | 0 |
| Total (maximum of 9) | 9 | 8 | 7 | 8 | 8 | 8 |
